# Supplementary material for: Relaxation Dynamics of Chlorophyll b in the Sub-ps Ultrafast Timescale Measured by 2D Electronic Spectroscopy
Source: Int J Mol Sci. 2020 Apr 18;21(8):2836. doi: 10.3390/ijms21082836 (PMC7215592; doi:10.3390/ijms21082836)
Supplement: Supplementary file 1 [file ijms-21-02836-s001.pdf]

SUPPORTING INFO

Relaxation dynamics of Chlorophyll *b* in the sub-ps ultrafast timescale measured by 2D electronic spectroscopy

Elisa Fresch,[1] Elisabetta Collini[1*]

[1] Department of Chemical Sciences, University of Padova, Via Marzolo 1, I-35131 Padova, Italy

Contents

S1. 2DES pulse characterization

S2. Additional 2DES data

S3. Raman spectrum of chl*b*

S1. 2DES pulse characterization

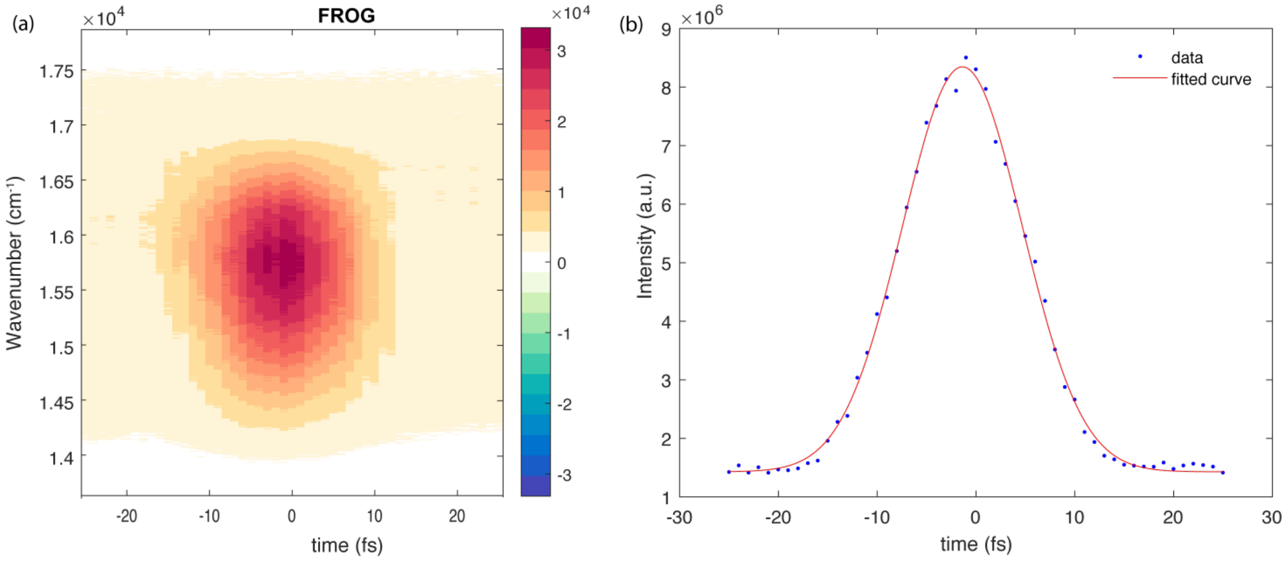

Figure S1. **(a)** FROG performed in the same experimental conditions of the 2DES experiments. **(b)** FROG signal integrated along the frequency axis. The experimental data (blue dots) are fitted by a gaussian function with FWHM of 14.3 (red curve), resulting in a pulse duration of 10.1 fs.

S2. Additional 2DES data

S2.1 Measures at room temperature

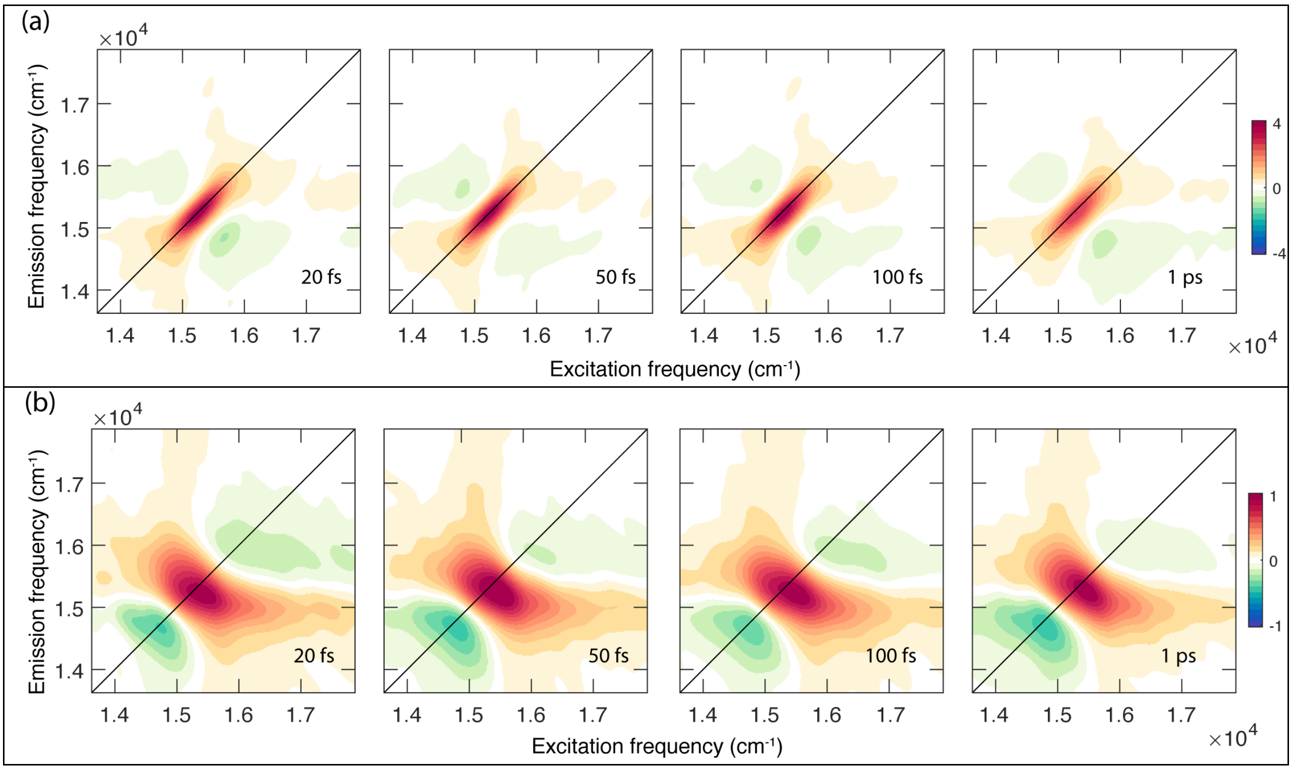

Figure S2.1. Evolution of **(a)** rephasing and **(b)** non-rephasing 2DES maps at selected values of population time t_2 for chl*b* at RT.

S2.2 Measures at 77K

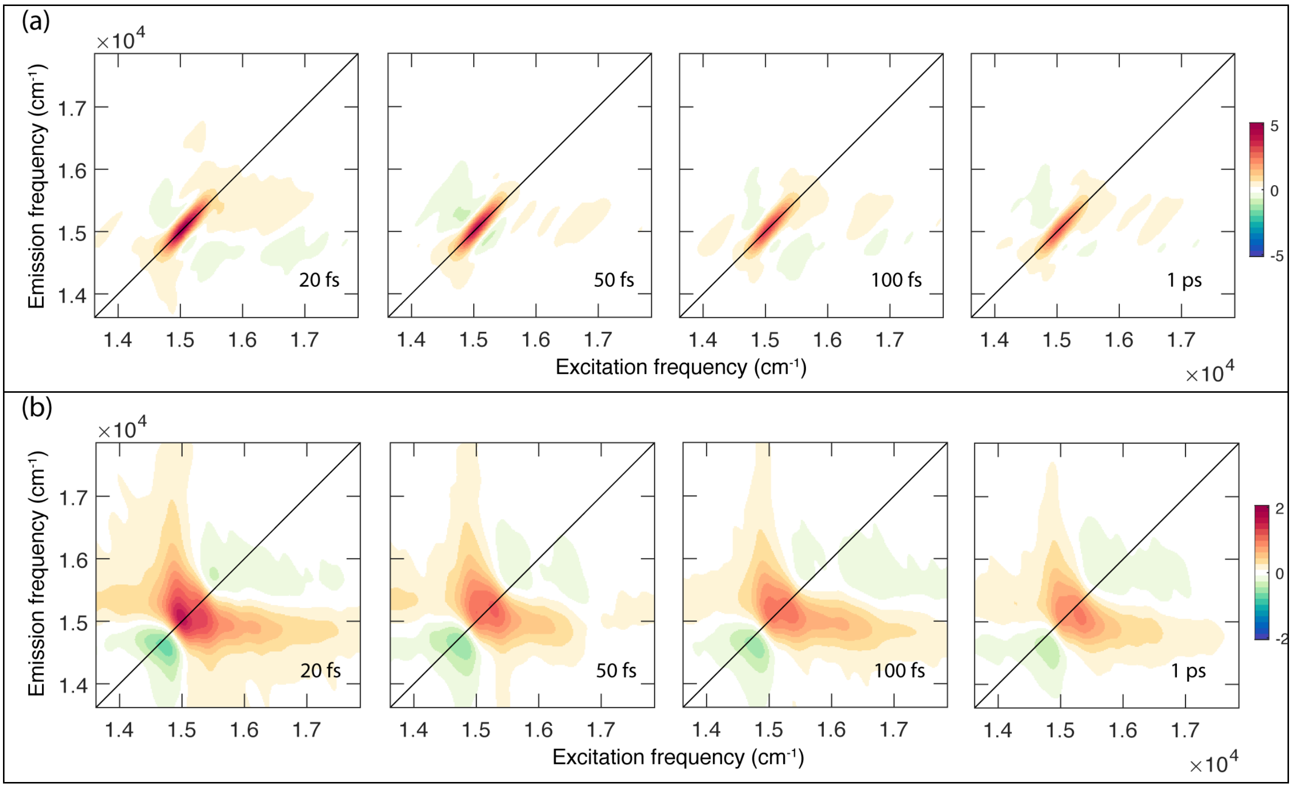

Figure S2.2. Evolution of **(a)** rephasing and **(b)** non-rephasing 2DES maps at selected values of population time t_2 for chl*b* at 77K.

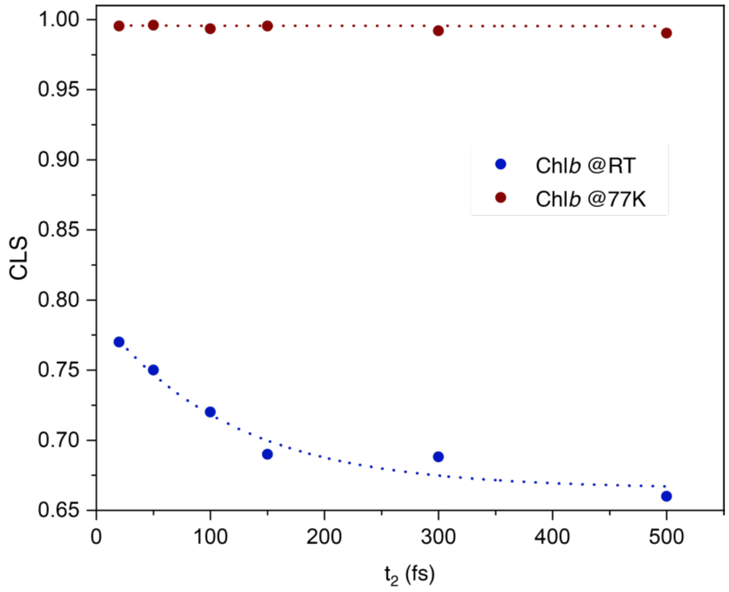

Figure S2.3. Centre line slope method results for chl*b* at RT (blue) and 77K (red). Following the procedure devised in ref. [Chem Phys 527, 110480, 2019], the center line of the 2D signal at a selected value of population time t_2 has been determined by using Gaussian functions fitted to slices parallel to the emission frequency *y*-axis. The obtained center line was then fitted by linear regression from which the angular coefficient was retrieved. We repeated this procedure for a number of 2D maps at selected values of t_2 from 20 to 500 fs and we plotted the slope values as a function of t_2 (circles). The CLS at RT clearly shows a mono-exponential decay behavior with a time constant of 115 ± 36 fs (blue dashed line). No decay of the CLS is instead recorded at 77K, implying that there is no correlation loss due to solvent dynamics in this timescale at this temperature. The error in the determination of the centre line slope is estimated in the order of the 10%.

S3. Raman spectrum of chl*b*

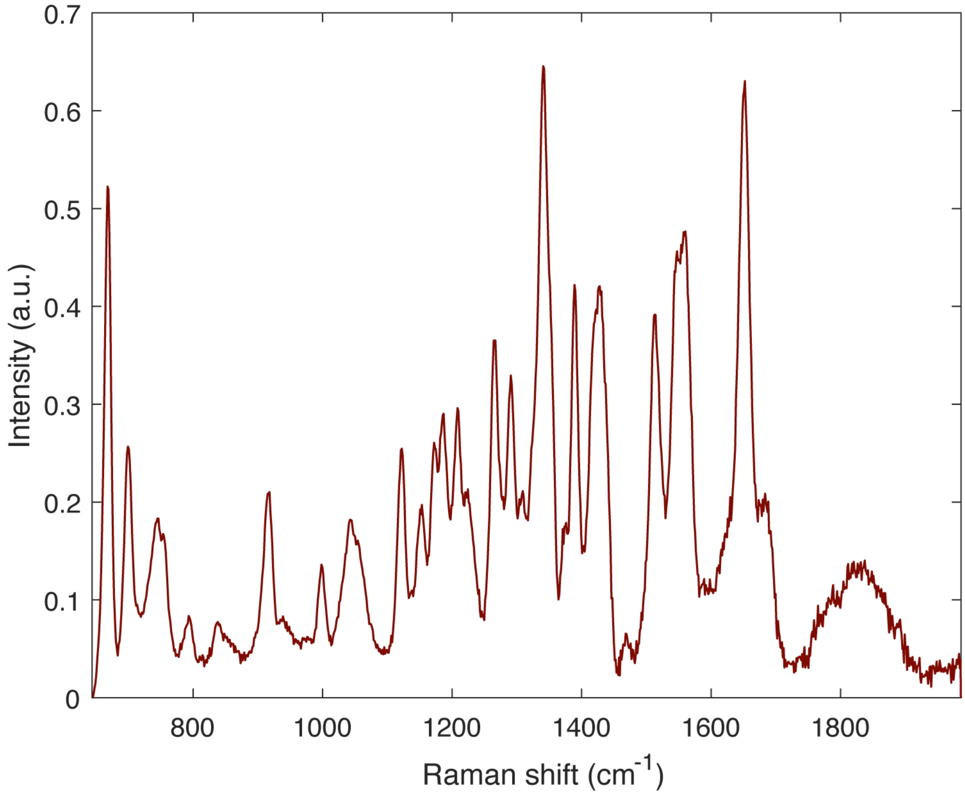

Figure S3. Raman spectrum of chl*b* powders recorded at an excitation wavelength of 488.0 nm.
